# An Acylsucrose-Producing Tomato Line Derived from the Wild Species *Solanum pimpinellifolium* Decreases Fitness of the Whitefly *Trialeurodes vaporariorum*

**DOI:** 10.3390/insects11090616

**Published:** 2020-09-09

**Authors:** María J. Rodríguez-López, Enrique Moriones, Rafael Fernández-Muñoz

**Affiliations:** Instituto de Hortofruticultura Subtropical y Mediterránea “La Mayora”, Universidad de Málaga-Consejo Superior de Investigaciones Científicas (IHSM, UMA-CSIC), Estación Experimental “La Mayora”, E-29750 Algarrobo-Costa, Malaga, Spain; mjrodriguez@eelm.csic.es (M.J.R.-L.); moriones@eelm.csic.es (E.M.)

**Keywords:** glandular trichomes, tomato, *Trialeurodes vaporariorum*, whitefly-resistance

## Abstract

**Simple Summary:**

The greenhouse whitefly, *Trialeurodes vaporariorum* is an insect pest of many plant crops including tomato and is especially harmful because it is a vector for a number of plant viral diseases. In this paper, an improved tomato line bred to produce glandular trichomes that exudate the deterrent compounds acylsucroses, which was introgressed from a wild tomato species, was demonstrated to decrease fitness of the insect and showed as a means for controlling the pests and, indirectly, could be an aid to reduce virus transmission to tomato plants.

**Abstract:**

A combination of biological control and host plant resistance would be desirable for optimally controlling the greenhouse whitefly, *Trialeurodes vaporariorum* in tomato crops. Whitefly settlement preference, oviposition, and survivorship were evaluated on ABL 10-4 and ‘Moneymaker’, two nearly-isogenic tomato lines with, and without, whitefly-resistance traits based on type IV leaf glandular trichomes derived from the tomato wild species *Solanum pimpinellifolium*, respectively. Significantly reduced preference of *T. vaporariorum* adult whiteflies for ABL 10-4 leaves was observed. Moreover, *T. vaporariorum* altered its abaxial–adaxial settling performance on leaves of ABL 10-4 plants. A significantly lower tendency to settle on abaxial leaf surface was observed in ABL 10-4 compared to Moneymaker plants. Furthermore, *T. vaporariorum* deposited fewer eggs and exhibited a significantly reduced egg to adult survivorship in ABL 10-4 than in Moneymaker plants. Therefore, reduced fitness and distorted performance were observed for *T. vaporariorum* on ABL 10-4 tomato plants supporting that type IV leaf glandular trichomes might protect them from this pest and, indirectly, from the viruses it transmits.

## 1. Introduction

The greenhouse whitefly, *Trialeurodes vaporariorum* Westwood (Hemiptera, Aleyrodidae), is a major insect pest of global importance in greenhouse-grown vegetables and ornamental cash crops. It is associated with damage to plants during feeding on plant phloem and by excreting honeydew that covers leaf foliage reducing transpiration and favoring the development of sooty mold, which reduces photosynthetic activity and crop quality [1,2,3]. However, the most serious damage caused by the greenhouse whitefly is the transmission of a number of viral diseases in different crop species [4]. In tomato plants (*Solanum lycopersicum* L.), *T. vaporariorum* is vector of plant viruses of the genera *Crinivirus* and *Torradovirus* (families Closteroviridae and Secoviridae, respectively) [4,5,6,7,8]. Among them, the crinivirus tomato chlorosis virus (ToCV) is a major emerging virus worldwide [4,9]. The direct and indirect damages caused by *T. vaporariorum* can then result in significant tomato yield losses [10,11].

Control of *T. vaporariorum* infestations is primarily dependent on the intensive use of insecticides, which disrupts natural biological control and can lead to the development of resistant insect populations [3,12,13] causing environmental and human health damage. Therefore, there is a need to develop more sustainable alternatives to control *T. vaporariorum*. Several biological control alternatives have been investigated extensively [14,15,16]. Host plant selection is the first stage of plant colonization by whiteflies and plays a major role in determining the evolution of whitefly populations in the field [17,18]. Plants have developed passive and active defenses to deal with pests by affecting their preference and/or performance [19,20]. Host plant resistance to insects can then be considered an interesting alternative to control insects in the framework of integrated pest management programs [21] and is gaining importance as increasing numbers of insecticides are being banned due to environmental and human health concerns. Host plant resistance to insect vectors is also one of the best strategies to manage circulative vector-borne virus diseases [22]. An advantage can then be gained from host plant resistance as an effective means to control whitefly infestations [23,24] which might help to reduce also incidence of transmitted viruses as demonstrated for the whitefly *Bemisia tabaci* Gennadius [25,26]. 

Plant resistance to arthropod herbivores is often mediated by phytochemicals that negatively affect the feeding, growth, or reproduction of the pest [27,28,29,30]. In this sense, wild relatives of tomato have been shown to possess effective means of dealing with several insect pests [28,31]. Resistance to the greenhouse whitefly has been reported in some wild tomato relatives such as *Solanum peruvianum* L., *Solanum habrochaites* S. Knapp & D.M Spooner, and *Solanum pennellii* Correll [24,32,33,34,35,36]. External barriers might be involved in whitefly preference for certain host plants. Factors on the leaf surface such as cuticle features and hairiness are known to influence host-plant selection. Glandular and nonglandular trichomes affect settling and survival of herbivores on host plants [37]. Volatile and non-volatile secondary metabolites are produced by glandular trichomes including acylsugars, terpenoids, phenylpropanoids, and flavonoids [37,38,39]. In *Solanum* species, type IV and type VI leaf glandular trichomes are associated with high levels of resistance to diverse arthropod species including mites, aphids, and whiteflies [36,40,41,42,43,44]. Early works in this area done by Gentile et al. [45] helped to identify resistance to *T. vaporariorum* in *S. hirsutum* (syn. *S. habrochaites*) and *S. pennellii* associated to a heavy vesture of sticky glandular exudates on leaves and stems. However, challenging the transference of resistance traits from the latter tomato green-fruited relatives to the cultivated tomato complicated the breeding process. Thus, linkage drag not easily removed by backcrossing results into commercially unfavorable alleles also transferred during this process. In recent times, whitefly resistance traits have been reported in wild tomato relatives with red fruits and more closely related to cultivated tomato that can facilitate their use. Thus, whitefly resistance was found in *Solanum galapagense* S. Darwin and Peralta and *Solanum pimpinellifolium* L. [25,31,33,46,47]. Interestingly, the traits found in *S. galapagense* involved in resistance to whiteflies were associated with the presence of type IV leaf glandular trichomes and the acylsugars they produce [41]. These trichomes and acylsugars, which are not present in cultivated tomatoes, also mediate the resistance of the wild species *S. pennellii* against many tomato pests [48]. Presence of type IV leaf glandular trichomes and acylsugar production was also reported in the accessions TO-937 of *S. pimpinellifolium* associated to arthropod resistance [40,49]. Moreover, the latter resistance trait demonstrated its effectiveness after successful introgression into the cultivated tomato providing antixenosis and antibiosis resistance to the *B. tabaci* whitefly and significant control of the worldwide damaging tomato yellow leaf curl virus (TYLCV) (genus *Begomovirus*, family Geminiviridae) [25,50].

*T. vaporariorum* is one of the most destructive whitefly species in field and greenhouse crops worldwide [51]. As the behavior of *T. vaporariorum* is known to be affected by plant volatile cues [52], here we investigated whether the presence of type IV leaf glandular trichomes and acylsugar secretion traits bred in domesticated tomato from *S. pimpinellifolium*, can help to deter this whitefly as it does with *B. tabaci* [25]. Therefore, we specifically assessed in the current study (i) the host-acceptance behavior and oviposition of *T. vaporariorum* in a tomato inbred line which presents type IV leaf glandular trichomes and acylsugar exudates, and (ii) life-history parameters such as egg and nymphal survival.

## 2. Materials and Methods 

### 2.1. Tomato Plants and Whitefly Population

Two near-isogenic tomato lines were used in this study, the whitefly- and virus-susceptible tomato cv. Moneymaker and ABL 10-4, an advanced backcross (BC_5_S_2_) line. For this BC_5_S_2_ line, presence of type IV leaf glandular trichomes and enhanced secretion of acylsucroses were derived from the initial cross *S. lycopersicum* cv. Moneymaker (without type IV leaf glandular trichomes) x *S. pimpinellifolium* accession TO-937 (with type IV leaf glandular trichomes, IHSM-CSIC germplasm collection) [44]. TO-937 is an inbred wild-tomato line derived from *S. pimpinellifolium* material collected by our colleague J. Cuartero at 50 m altitude on the coastal plain of Lambayeque Department, Peru, in 1983. The original accession segregated widely for density of glandular trichomes and was fixed by four consecutive selfing and selection steps. Obtaining the BC_5_S_2_ line AB 10-4 involved five cycles of combined recurrent crosses toward Moneymaker and subsequent selfing steps with selection for high type-IV leaf trichome density and acylsucrose production, plus two additional final selfing steps [53].

Plantlets of ABL 10-4 and tomato cv. Moneymaker were individually sown in pots (30 cm diameter) containing a mixture of 50% soil (54% sand, 24% silt, and 22% clay), 30% horticultural substrate, 15% coconut–fiber substrate and 5% litonite (loaded zeolite). Until used, plants were grown in an insect-proof glasshouse with temperature control (22 to 27 °C day and 17 to 20 °C night) and supplied weekly with nutrient solution. Experiments were conducted taking into account the plant growth stage because significant difference in acylsucrose production between Moneymaker and an advanced backcross lines with type IV leaf glandular trichomes was only achieved after the ten-leaf stage [25].

For the experiments, non-viruliferous *T. vaporariorum* whiteflies were reared on melon (*Cucumis melo* L. ‘ANC42’, IHSM-CSIC germplasm collection) plants within wooden cages covered with insect-proof nets, in an insect-proof glasshouse with temperature control (22 to 27 °C day and 17 to 20 °C night) and supplemental light when needed. The initial whitefly colony originated from *T. vaporariorum* field individuals collected in Malaga (Spain).

### 2.2. Trichome Observation and Acylsucrose Accumulation Quantification

Type IV trichomes are located on both the adaxial and abaxial sides of the leaf but, in a BC_3_S_2_ line also derived from TO-937, they are far more abundant on the abaxial than on the adaxial leaf surface [54]. Type IV trichome density of ABL 10-4 and Moneymaker was measured following the indications by Alba et al. [40]. Previous analysis of TO-937 and the derived *S. lycopersicum* introgression lines indicated that these produced sucrose esters [40]. Epicuticular leaf acylsucroses were then extracted and de-esterified using the method described by Goffreda et al. [55], and the resulting free-sugar moiety was quantified spectrophotometrically using a hexokinase-based glucose assay. In short, aliquots of acylsucroses were concentrated by evaporation, re-dissolved in methanol and saponified adding 0.04N NaOH. Free sucrose was hydrolyzed to glucose and fructose by adding invertase (Sigma-Aldrich, St. Louis, MO, USA, ref. I9274), and then phosphorylated by adenosinetriphosphate (ATP, Sigma-Aldrich, St. Louis, MO, USA, ref. A26209) in the reaction catalyzed by hexokinase (Sigma-Aldrich, St. Louis, MO, USA, ref. H6389). The resulting glucose-6-phosphate was oxidized to 6-phosphogluconate in the presence of nicotinamide adenine dinucleotide phosphate (NADP, Sigma-Aldrich, St. Louis, MO, USA, ref. N5755) in a reaction catalyzed by glucose-6-phosphate dehydrogenase (Sigma-Aldrich, St. Louis, MO, USA, ref. G6378). The absorbance at 340 nm was recorded, and sucrose quantities were determined by using a sucrose standard curve in the range of 0.15–5.8 mM, and expressed as nmol of sucrose esters per cm^2^ of leaf area. To normalize the data and stabilize the variance, trichome and acylsucrose measurements were Log (x + 1) transformed prior to analysis. Statistical differences between the means of trichome IV density and acylsucrose production in the two genotypes were analysed by one-way ANOVA and the Fisher’s least significant difference (LSD) test by using IBM SPSS Statistics for Windows, Version 26.0 (IBM Corp., Armonk, NY, USA).

### 2.3. T. vaporariorum Settling Preference

Whitefly settling behavior of adult individuals of *T. vaporariorum* was assessed on the adaxial and the abaxial leaflet surfaces of ABL 10-4 and the whitefly-susceptible cv. Moneymaker tomato plants under non-choice conditions following the experimental design described by Rodriguez-Lopez et al. [25]. Detached leaflets from Moneymaker and ABL 10-4 tomato plants at ten-leaf growth stage were used. Six leaflets of one genotype were placed forming a circle in an independent methacrylate box (22 × 22 × 7 cm). Each leaflet petiolule was inserted in a plastic dish (2 cm diameter × 1 cm high) filled with nutrient solution (0.25 g/L of Nutrichem 60, Miller Chemical, Hanover, PA, USA) to maintain leaflet turgor. Leaflets were placed abaxial surface down and at an angle with the horizontal (so that both leaflets surfaces were freely accessible to whiteflies), with their tips directed to the center of the circle formed by leaflets. Thirty adult whiteflies (five whiteflies per leaflet tested, without sex distinction) were released in the center of the circle after a short cold treatment (10 min at 4 °C) to facilitate handling. Each methacrylate box was then covered with a methacrylate lid with an opening covered with muslin for ventilation. The boxes were placed in a growth chamber (26 °C day and 22 °C night, 70% relative humidity, with a 16-h photoperiod at 250 mol·s^−1^·m^−2^ photosynthetically active radiation). The number of whiteflies that settled on both surfaces of leaflet was counted at 0.5, 1, 2, 4, 8, 24, and 48 h after release. Each experiment was replicated 12 times. The mean number of whiteflies per leaflet and leaflet surface on each genotype were calculated. Settling preferences were statistically analyzed by Generalized Linear Models (GzLM) with Log as the link function and Poisson as the probability distribution, and the means of the two genotypes at each time point were compared by the least-squares (LS) means test by using the IBM SPSS Statistics package. 

### 2.4. Oviposition of T. vaporariorum

A non-choice test was conducted to compare the oviposition rates of *T. vaporariorum* on leaves of the two near-isogenic tomato lines cv. Moneymaker and ABL 10-4. Five adult females of *T. vaporariorum* were selected from the rearing colonies with the help of a stereomicroscope (40×) and placed into a leaf-clip cage (five whiteflies per clip-cage). Previously, whiteflies received a cold treatment (at 4 °C for 10 min) to reduce their activity and facilitate handling for sex distinction. The leaf-clip cages were attached to the abaxial leaflet surface of the fourth newest leaf from the growing point of each test plant (at ten-leaf growth stage). The number of eggs deposited was counted after 24 h using a manual lens (20×) and the mean number of eggs per female was calculated. The experiment included fifteen replicates for the whitefly-susceptible tomato cv. Moneymaker and twenty replicates for ABL 10-4. The adaxial leaf side was not assayed because in our previous observations when developing the inbred lines, whitefly oviposition was never observed to occur on Moneymaker or the improved advanced-backcross lines. Data were analyzed by GzLM with Log as the link function and Poisson as the underlying distribution, and means from the two genotypes were compared by the LS means test by using the IBM SPSS Statistics package.

### 2.5. Survival of Immature T. vaporariorum

Five adult females of *T. vaporariorum* were confined into leaf-clip cages as described above. The leaf-clip cages were then attached to the abaxial leaflet surface of the fourth newest leaf from the growing point of each test plant (at ten-leaf growth stage) of Moneymaker and ABL 10-4. The adult whiteflies were removed after 24 h and the remaining founder eggs and nymphs were monitored over the following 33 days until nymphs developed to L4 stage [56] in the whitefly-susceptible Moneymaker cultivar. Analysis of survival time was estimated by the Kaplan–Meier estimator of the survivorship function [57]. Comparison of survivorship functions was made using the generalized Wilcoxon test as described by Hosmer and Lemeshow [58].

## 3. Results

### 3.1. ABL 10-4 Accumulates Type IV Glandular Trichomes and Acylsucroses in Leaves

**Figure 1:**
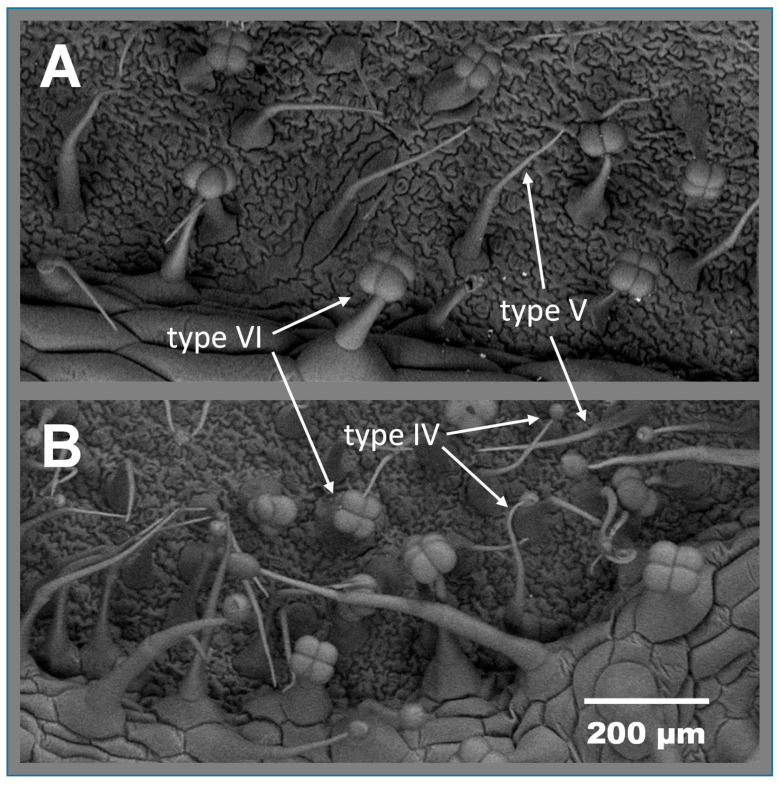
Scanning electron micrographs of abaxial leaf surfaces from (**A**) ‘Moneymaker’ and (**B**) the acylsucrose-producer breeding line ABL 10-4. Type IV glandular trichomes are only present in ABL 10-4 while nonglandular type V and glandular type VI trichomes are present in the two genotypes.

Successful introgression of acylsucrose-producing type IV leaf glandular trichomes in Moneymaker background was achieved in the advanced backcross line (BC_5_S_2_) ABL 10-4 (Figure 1B) while no such glandular trichomes are present in the background genotype (Moneymaker) plants (Figure 1A).

**Figure 2:**
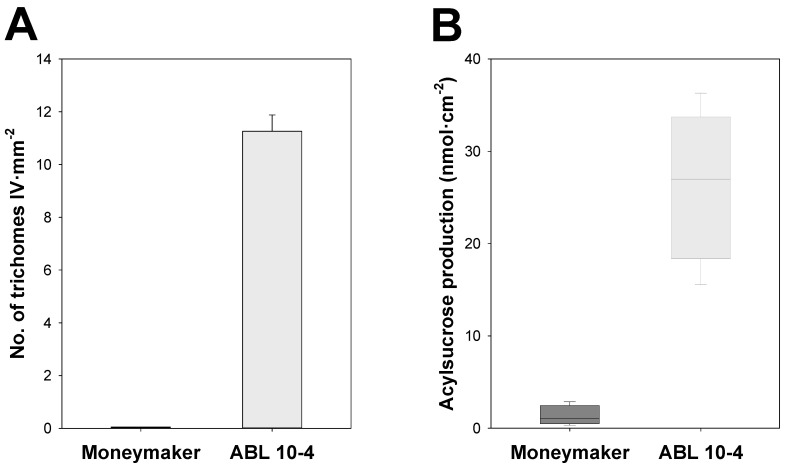
Type IV leaf trichome density and production of acylsucrose in Moneymaker and ABL 10-4 near-isogenic tomato lines. (**A**) Bar graph showing type IV leaf glandular trichome density on abaxial leaflet surfaces of Moneymaker and ABL 10-4 plants at the ten-leaf growth stage. (**B**) Box-and-whisker plots showing acylsucrose accumulation on leaflets of Moneymaker and ABL 10-4 plants at the ten-leaf growth stage; the box represents the interquartile range, the horizontal line in the box shows the value of the median, and bars mark the 10th and 90th percentiles.

As a result, tomato cv. Moneymaker and its near-isogenic line ABL 10-4 strongly differed for type IV leaf glandular trichome-associated traits. In ABL 10-4, type IV leaf glandular trichomes density was around 11 trichomes per mm^2^ on the abaxial leaflet surface (Figure 2A). Consequently, a significantly enhanced secretion of acylsucroses was detected in ABL 10-4 compared to Moneymaker plants (*p* = 0.001, LSD test) (Figure 2B). 

### 3.2. T. vaporariorum Repellence in the Genotype with Type IV Glandular Trichomes

**Figure 3:**
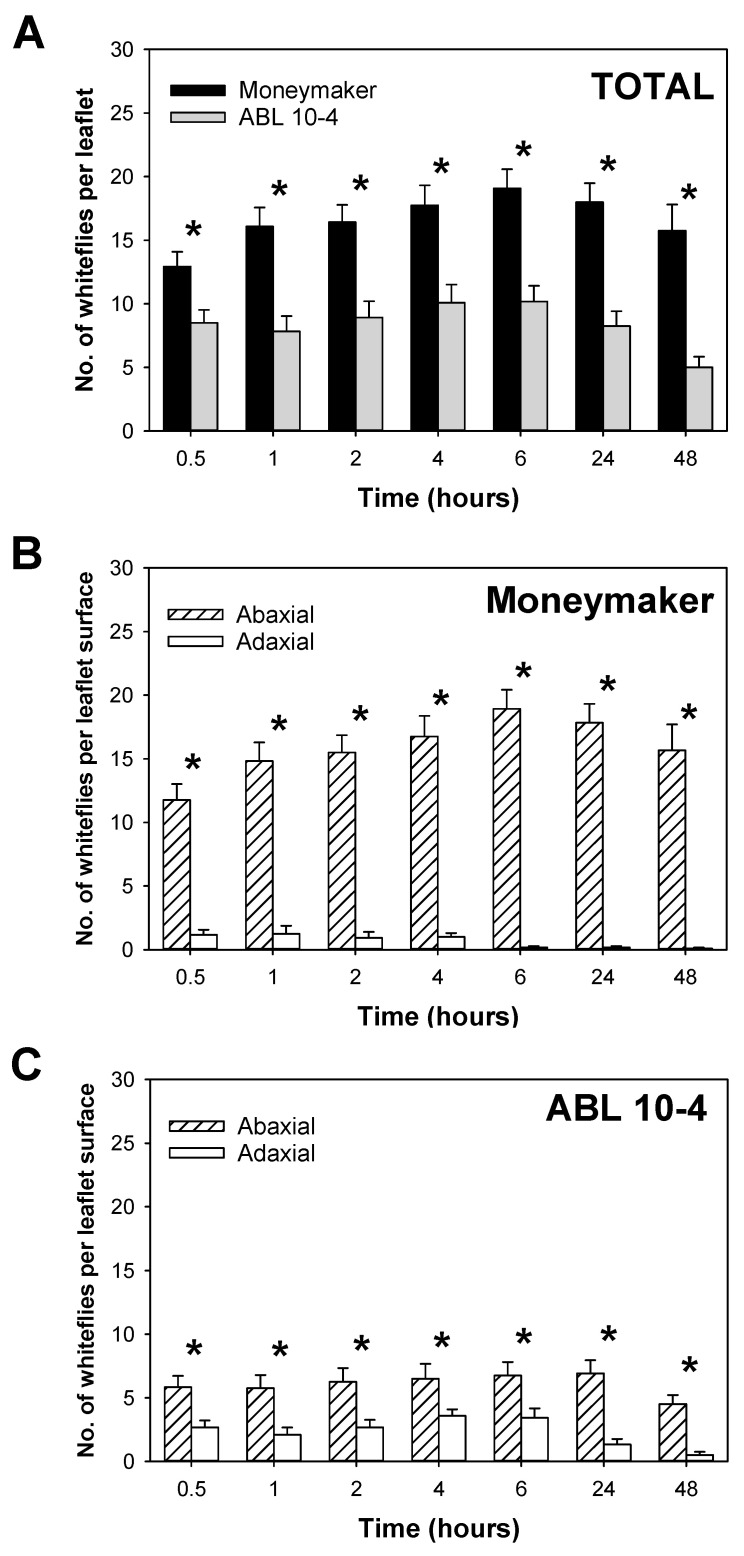
*Trialeurodes vaporariorum* settling on leaves of Moneymaker and ABL 10-4 near-isogenic tomato lines in non-choice conditions calculated at different times after the release of five *T. vaporariorum* adult whiteflies on Moneymaker and ABL 10-4 test leaflets at the center of arena. (**A**) Total number of whiteflies settling on any of the leaf surfaces of Moneymaker vs. ABL 10-4, (**B**) number of whiteflies on abaxial vs. adaxial leaf surface of Moneymaker, and (**C**) on abaxial vs. adaxial leaf surface of ABL 10-4. Asterisks indicate significant differences (*p* = 0.05) at each time point based on LS means test under generalized linear models (Log link, Poisson distribution) analyses; bars indicate the standard error of the mean.

In the repellence experiments, no significant number of dead whiteflies was detected in any treatment. Under the non-choice conditions tested, a significantly (*p* = 0.001, LS means tests) lower settling preference (antixenosis) of *T. vaporariorum* was observed at all time points on the leaflets of the acylsucrose-producing genotype ABL 10-4 when compared to Moneymaker (Figure 3A). When counted separately, significantly higher mean number of whiteflies settled on Moneymaker than on ABL 10-4 for the abaxial leaf side while the opposite was observed for the adaxial surface (data shown in Figure 3B,C, statistically analysed as in Figure 3A). Regarding leaf sides comparisons in each plant line, a significantly (*p* = 0.001, LS means test) higher mean number of whiteflies was counted on the abaxial than on the adaxial side of Moneymaker leaflets since early times after release (Figure 3B). Moreover, significantly higher (*p* = 0.001, LS means test), but less pronounced differences in the mean number of whiteflies that settled on abaxial than on adaxial leaflet surfaces at every time evaluated were observed in ABL 10-4 (Figure 3C). Taken together, results indicated that *T. vaporariorum* exhibits a modified settling performance on ABL 10-4 plants; the abaxial side of leaves which is the ideal settling site for this whitefly species, was not so clearly preferred by the insect when exposed to leaflets from the type IV-bearing plant genotype.

### 3.3. Negative Effect of ABL 10-4 on Oviposition and Survival of T. vaporariorum

**Figure 4:**
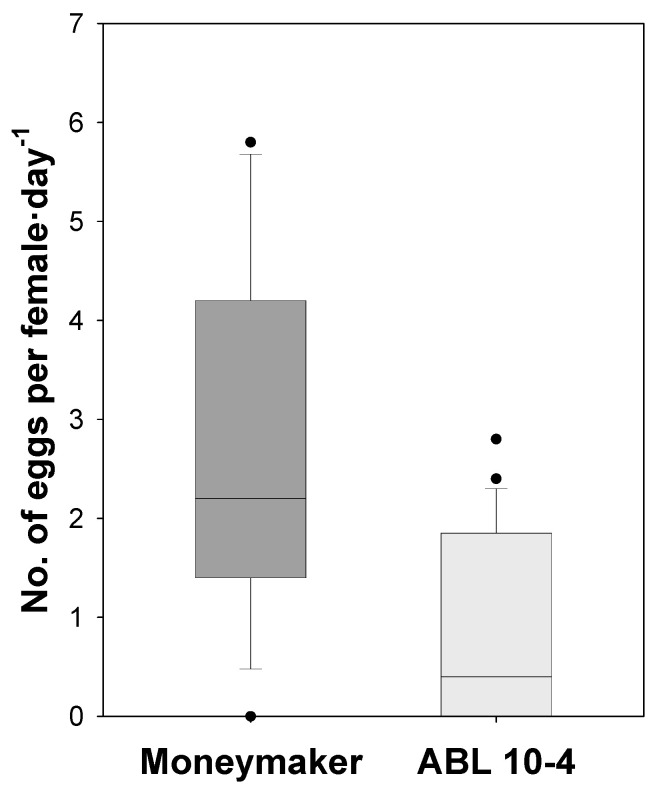
Oviposition rates of *Trialeurodes vaporariorum* on Moneymaker and ABL 10-4 near-isogenic tomato lines. Box-and-whisker plots show the number of eggs per female of *T. vaporariorum* counted on leaf area of clip-cage (1.5 cm Ø) exposed to whiteflies at 24 h after whitefly release. The box represents the interquartile range, the horizontal line in the box shows the value of the median, bars mark the 10th and 90th percentiles, and dots show outlier values.

Presence of acylsucrose-producing type IV leaf glandular trichomes in ABL 10-4 not only reduced *T. vaporariorum* settling but also prevented its oviposition. On ABL 10-4 leaves, *T. vaporariorum* laid significantly (*p* = 0.001) lower mean number of eggs per female (0.77 ± 0.20) than on leaves of tomato cv. Moneymaker (2.47 ± 0.46) plants (Figure 4). Therefore, the significantly reduced number of eggs deposited on leaves of ABL 10-4 plants suggested that the presence of type IV acylsucrose-producing trichomes reduced oviposition acceptance of *T. vaporariorum*.

**Figure 5:**
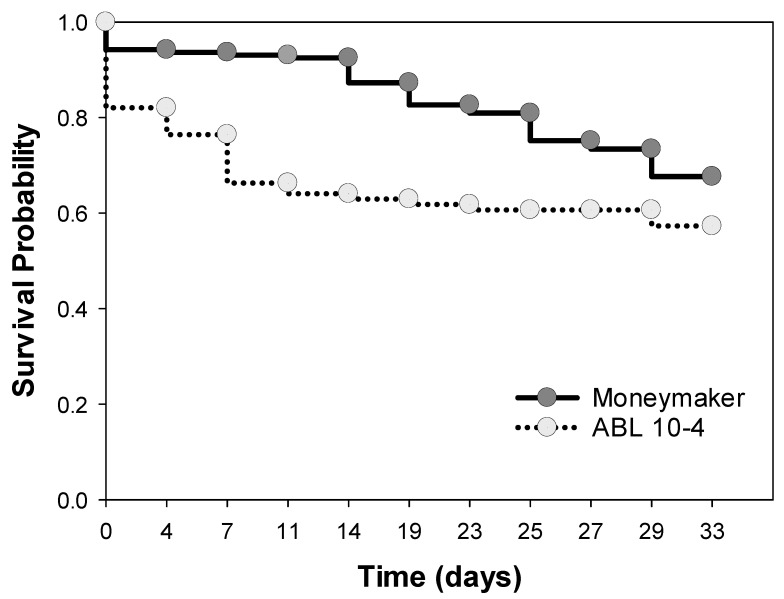
*Trialeurodes vaporariorum* survival probability on plants of Moneymaker and ABL 10-4 near-isogenic tomato lines during a period of 33 days after release. Survival time of *T. vaporariorum* on leaves of the lines was estimated based on the Kaplan–Meier estimator of the survivorship function [57]. Comparison of survivorship functions was performed by using the test described by Hosmer and Lemeshow (1999).

Moreover, a significant negative effect was observed on the survival of *T. vaporariorum* immature stages on ABL 10-4 compared to Moneymaker. As shown in Figure 5 that represents the survival probability for immature stages of whiteflies from egg (day 1) to L4 nymph (day 33), survival was significantly (*p* = 0.001; generalized Wilcoxon test) lower on ABL 10-4 than on Moneymaker leaves. Difference in survival probability was highly accentuated at early stages previous to hatching of eggs (about day 11). From day 11 on, the survival probability of *T. vaporariorum* immature stages on Moneymaker declined to 69% by day 33, whereas in ABL 10-4 was almost constant with a final decline to 59% by day 33. Therefore, the negative effect of the presence of type IV acylsucrose-producing trichomes on immature stages survival is mostly associated with reduced egg survival, rather than on larval survival that, for later stages seemed to be higher on leaves of ABL 10-4.

## 4. Discussion

A novel approach to global pest management is the use of phytochemicals based on organic chemical compounds that are repellent, with the deterrent effect resulting into reduced insect attractiveness of the plant. Here it was demonstrated that the antixenosis and deterrent traits from *S. pimpinellifolium* found effective against to *B. tabaci* [25] were also effective against *T. vaporariorum*.

The results showed that *T. vaporariorum* adults were deterred by the type IV leaf glandular trichomes and acylsucroses present on ABL 10-4 leaf surface. The association between insect resistance and the presence and density of type IV leaf glandular trichomes has been reported in *S. habrochaites* and *S. pennellii* by several authors [42,55,59,60,61]. Goffreda et al. [42] described that acylsugars in the exudates of type IV trichomes in *S. pennellii* mediated the resistance to the potato aphid by deterring insect settling and phloem feeding. Moreover, Maluf et al. [48] demonstrated that the foliar acylsugars content was a major component of the resistance to three tomato pests (including the whitefly *B. tabaci*) in tomato genotypes with type IV leaf glandular trichomes derived from *S. pennellii*. Foliar acylsugars from type IV leaf glandular trichomes in *S. pimpinellifolium* TO-937 (the resistance donor of the ABL 10-4 tomato line), were also shown to be sufficient to confer resistance to the two-spotted spider mite *Tetranychus urticae* Koch (Prostigmata:Tetranychidae) and the whitefly *B. tabaci* [25,40,44]. Results shown here demonstrated that as for *B. tabaci* in previously reported studies [54], *T. vaporariorum* exhibited an altered settling behavior in abaxial and adaxial sides of ABL 10-4 leaves. Most pierce-sucking hemipterans including *B. tabaci* whiteflies prefer to settle, feed, and oviposit on the abaxial than on the adaxial leaf surface of their host plants soon after landing [43,62,63]. However, a significantly less pronounced preference for the abaxial side was observed for *T. vaporariorum*, associated with an increased settling on the adaxial side of leaves of ABL 10-4 compared to Moneymaker. These observations demonstrated that the abaxial surface of ABL 10-4 deterred *T. vaporariorum* settling in contrast to what was observed for leaves of the whitefly-susceptible Moneymaker. Interestingly, in our observations during the preference experiments of this present work and during the previous work at greenhouse cultivations of the plants for selecting the acylsucrose-producing plants, we never detected oviposition on the adaxial leaf sides of either plant genotype. Therefore, ABL 10-4 genotype modifies the innate behavior of *T. vaporariorum* for settling and feeding on the abaxial surface of tomato leaves. This altered behavior is likely due to the presence of deterrent acylsugars secreted by the type IV leaf glandular trichomes, which are mainly located on the abaxial leaflet surface of the whitefly-resistant genotype [54]. Similar deterrent effect was observed against *T. urticae* by TO-937 (the type IV leaf glandular trichomes donor of ABL 10-4) which related mostly to abaxial density of type IV leaf glandular trichomes [44]. Therefore, in contrast to that observed in Moneymaker, *T. vaporariorum* exhibited difficulties settling on abaxial leaflet surface when exposed to ABL 10-4 and an increased number of whiteflies settle on the adaxial side of the leaf. Consequently, the *T. vaporariorum* tendency to locate preferentially on the abaxial surface of plant leaves, not easily reached by conventional insecticide spraying equipment [13] is altered in ABL 10-4. This altered behavior in the latter tomato genotype might help to increase *T. vaporariorum* control when using conventional insecticide spraying. Furthermore, when moving to the adaxial leaf surface, *T. vaporariorum* will become more exposed and then more vulnerable to the action of natural enemies. Better predator activity on adaxial side of leaves was reported, e.g., for *Delphastus catalinae*, an important coccinellid predator of *B. tabaci* [64].

Presence of acylsucrose-producing type IV leaf glandular trichomes in ABL 10-4 not only reduced *T. vaporariorum* settling but also prevented its oviposition. In fact, a similar oviposition reduction effect was also observed for the whitefly *B. tabaci* in *S. pennellii* [65] or in the *S. pimpinellifolium* accession TO-937, the insect-resistance donor of ABL 10-4 [66]. Moreover, presence of acylsugars has been shown to be associated to insect resistance traits in several wild tomato relatives [41,48] also altering oviposition behavior [67,68]. A significant detrimental effect was also observed here for the survival of *T. vaporariorum* immature stages on ABL 10-4 compared to Moneymaker, mostly associated with egg survival. In this sense, Buta et al. [69] described the isolation and identification of a group of sucrose esters from *Nicotiana gossei* active against the immature stages of *T. vaporariorum* when applied topically. Similar to our results, Bas et al. [24] described that the resistance to *T. vaporariorum* in *S. habrochaites* f. *glabratum* is associated to a low pre-adult survival. Our results on immature stages survival as another component of the *T. vaporariorum* resistance in ABL 10-4 are coincident with previous observations [70,71] that associated the mortality of *T. vaporariorum* in a number of plant species including tomato to reduced survival of eggs and first developmental stages. Apparently, late instar nymph survival was higher on ABL 10-4 than on Moneymaker plants. This may be due to true better fitness of late instar nymphs in an acylsucrose-rich plant environment but this could also be an artifact caused by biased nymphal populations in the two genotypes. Biased nymphal populations may be produced by adaptation to the acylsucroses of the nymphs that survived the first developmental stages on ABL 10-4 leaves, or by existence of strong bottleneck selection of whitefly genotypes eventually more tolerant to acylsucrose detrimental effects. More precise experiments with nymphs from the different developmental stages individually located on the leaves of the two plant lines would be required to elucidate this aspect.

This present study demonstrates that the insect resistance based on type IV leaf glandular trichomes and acylsucrose secretions derived from *S. pimpinellifolium* TO-937 and introgressed in a nearly-isogenic BC_5_S_2_ tomato line ABL 10-4 can be used as a sustainable control approach to reduce the fitness of the greenhouse whitefly *T. vaporariorum* on this crop. Transfer of phytochemical-mediated resistance to cultivated tomato species as a means to control multiple pests could significantly reduce the use of agrochemical sprays [28,29,31,68,72]. Previous studies on control of whiteflies based on acylsugar secretions bred from the wild tomato *S. pennellii* [68,73,74] resulted in sticky plant materials whose use might interfere with the effectiveness of natural enemies used in biological control programs [75,76]. No such unwanted stickiness was observed in the tomato line ABL 10-4 (unpublished observations) that might interfere with biological control of pests. In a previous work we reported that acylsucrose-producing type IV leaf glandular trichomes from *S. pimpinellifolium* resulted in a reduced preference and affected the feeding behavior of the whitefly *B. tabaci* that were effective to reduce the spread of TYLCV [25], a virus transmitted by this whitefly. The results of the current study for *T. vaporariorum* together with the previous results regarding *B. tabaci*, suggest that the tomato isogenic lines derived from *S. pimpinellifolium* that exhibit type IV leaf glandular trichomes and acylsucrose secretion might be a promising tool for resistance to either *B. tabaci* and *T. vaporariorum* whiteflies in tomato breeding programs. Conventional plant breeding could then contribute to the control of these whiteflies and the viruses they transmit in tomato. Nevertheless, as the effect of vector-resistance on virus spread might depend on a number of factors like, among others, the length of the acquisition, retention, inoculation and latency periods of the virus, the possible existence of non-linear relationship between number of vectors and virus transmission, and the potential enhanced mobility of vector insects in the repellent plants that could eventually increase virus spread, further investigation will be needed to evaluate the possible benefits of using the described *T. vaporariorum*-resistance as a mean to control the viruses it transmits such as criniviruses or torradoviruses [6].

## 5. Conclusions

This study demonstrates that the insect resistance based on type IV leaf glandular trichomes and acylsucrose secretions introgressed from a wild tomato into the cultivated species reduces the fitness of the greenhouse whitefly *T. vaporariorum*. Improved plant materials producing acylsucroses can be useful for sustainable control of this vector insect of several viral diseases affecting tomato crop.
